# Deep sequencing of biofilm microbiomes on dental composite materials

**DOI:** 10.1080/20002297.2019.1617013

**Published:** 2019-05-14

**Authors:** Georg Conrads, Laura Katharina Wendt, Franziska Hetrodt, Zhi-Luo Deng, Dietmar Pieper, Mohamed M. H. Abdelbary, Andree Barg, Irene Wagner-Döbler, Christian Apel

**Affiliations:** a Division of Oral Microbiology and Immunology, Department of Operative and Preventive Dentistry & Periodontology, RWTH Aachen University Hospital, Aachen, Germany; b Department of Biohybrid & Medical Textiles, Institute of Applied Medical Engineering, RWTH Aachen University, Aachen, Germany; c Group Microbial Communication, Helmholtz Center for Infection Research (HZI), Braunschweig, Germany; d Group Microbial Interactions and Processes, Helmholtz Center for Infection Research (HZI), Braunschweig, Germany; e VOCO GmbH, Cuxhaven, Germany

**Keywords:** Microbial ecology, biofilm, microbiome, *Streptococcus mutans*, composite materials, bovine enamel, caries, carolacton

## Abstract

**Background**: The microbiome on dental composites has not been studied in detail before. It has not been conclusively clarified whether restorative materials influence the oral microbiome.

**Methods**: We used Illumina Miseq next-generation sequencing of the 16S V1-V2 region to compare the colonisation patterns of bovine enamel (BE) and the composite materials Grandio Flow (GF) and Grandio Blocs (GB) after 48 h *in vivo* in 14 volunteers. Applying a new method to maintain the oral microbiome *ex vivo* for 48 h also, we compared the microbiome on GF alone and with the new antimicrobial substance carolacton (GF+C).

**Results**: All *in vitro* biofilm communities showed a higher diversity and richness than those grown *in vivo* but the very different atmospheric conditions must be considered. Contrary to expectations, there were only a few significant differences between BE and the composite materials GB and GF either *in vivo* or *in vitro: Oribacterium, Peptostreptococcaceae* [XI][G-1] and *Streptococcus mutans* were more prevalent and *Megasphaera, Prevotella oulorum, Veillonella atypica, V. parvula, Gemella morbillorum*, and *Fusobacterium periodonticum* were less prevalent on BE than on composites. *In vivo*, such preferences were only significant for *Granulicatella adiacens* (more prevalent on BE) and *Fusobacterium nucleatum* subsp. *animalis* (more prevalent on composites). On DNA sequence level, there were no significant differences between the biofilm communities on GF and GF+C.

**Conclusion**: We found that the oral microbiome showed an increased richness when grown on various composites compared to BE *in vitro*, but otherwise changed only slightly independent of the *in vivo* or *in vitro* condition. Our new *ex vivo* biofilm model might be useful for pre-clinical testing of preventive strategies.

## Introduction

The concept of aesthetic and minimally invasive dentistry has led to the preferred use of direct restorative materials. However, light-curing composite materials possess special characteristics that may reduce their lifespan compared to other filling materials such as amalgam and gold. The clinical success of such composites is hindered by shrinkage, technique sensitivity, and the absence of antibacterial properties. Restoration longevity is closely linked to susceptibility towards bacterial colonization. There is growing evidence that complex bidirectional interactions between the biodegradation of composite resins and bacterial colonization (with acid production and lower pH levels) increases the rate of secondary caries, the most frequent reason for restoration failure [1,2]. Saliva and oral bacteria such as *Streptococcus mutans* display esterase activity, which accelerate the breakdown of the resin–tooth interface [3,4]. The development of strategies to enhance the stability of composite resins within the oral cavity therefore requires detailed information about bacterial colonisation. Bacteria adhere more strongly to composites than other dental materials [5,6] but the qualitative and quantitative shift in bacterial taxa *in vivo* has not been investigated in detail. Streptococci play a major role in plaque biofilm formation, with the highly cariogenic species *S. mutans* involved in the later stages of this process [7,8]. The biostability of dental materials can be increased by inhibiting bacterial adhesion [1]. For example, carolacton is a macrolide keto-carboxylic acid produced by the myxobacterium *Sorangium cellulosum* and was shown to reduce the viability of *S. mutans* biofilm cells [9,10]. We have previously incorporated carolacton into a composite resin and demonstrated a significant biofilm-damaging effect *in vitro* [11]. Here we used Illumina next-generation sequencing to analyse the biofilm microbiome attached to composite materials worn *in vivo* by human volunteers. We also collected clinically relevant information about the potential antimicrobial effect of carolacton on complex oral communities. Because carolacton is not approved as a drug, we developed a new method for the ex vivo maintenance of biofilms from the saliva of the same volunteers, and tested the influence of carolacton using this model. Our study is the first to identify and compare biofilm microbiomes on composite materials *in vivo* and *in vitro*.

## Materials and methods

### Ethical aspects and inclusion/exclusion criteria

The study protocol was approved by the local institutional ethical committee (Medical Faculty, RWTH Aachen University, Germany) under reference EK336/16. The 14 volunteers (8 women and 6 men, aged 22–53 years) signed a written informed consent form before screening. The participants met all the following inclusion criteria: (1) the ability to wear a mandibular appliance for 48 h, except for food intake and normal daily tooth brushing; (2) evidence of increased caries risk with a decayed, missing and filled teeth (DMFT) index of 1–21; (3) the existence of cariogenic flora based on a positive real-time quantitative PCR test for the presence of *S. mutans* and lactobacilli [12]. The exclusion criteria were antibiotic medication during the past 4 weeks, smoking, noncompliance with the study procedures, age <18 years, and no written informed consent/inability to form a contract.

### Specimen preparation

The composites Grandio Flow (GF) and Grandio Blocs (GB) were supplied by Voco GmbH, Germany. GF is a commercial light-curing flow composite and GB is a computer-aided design and computer-aided manufacturing (CAD/CAM) material without photo-initiators, which is chemically cured under high pressure. GF incorporating 50 µg/ml carolacton (named GF+C) was produced as previously described [11]. Composite specimens (5 × 1.5 mm) were prepared with using a moulding form under sterile conditions. GF and GF+C were light-cured under a glass slide to avoid an oxygen-inhibited layer using a bluephase C8 device (Ivoclar Vivadent, Germany) and the specimens were used without further polishing. GB was prepared using a CEREC dental CAD/CAM system (Dentsply Sirona, Germany), polished with Phoenix Alpha abrasive paper (FEPA grit size P#800, P#1200, P#2400, P#4000 silicon carbide, supplied by Wirtz-Buehler, Germany) and disinfected with 70% ethanol. Bovine enamel (BE) control samples were prepared from freshly extracted bovine incisors stored in 5% thymol at 4°C. The teeth were cut into blocks using an Exakt 300 (Exakt Apparatebau, Germany), ground flat on the enamel side and hand polished as above to produce 5 × 1.5 mm samples. All samples were rinsed, stored in physiological saline solution and autoclaved (136°C for 15 min) before each experiment.

### Overall study design

#### In vivo intraoral phase

**Figure 1.**
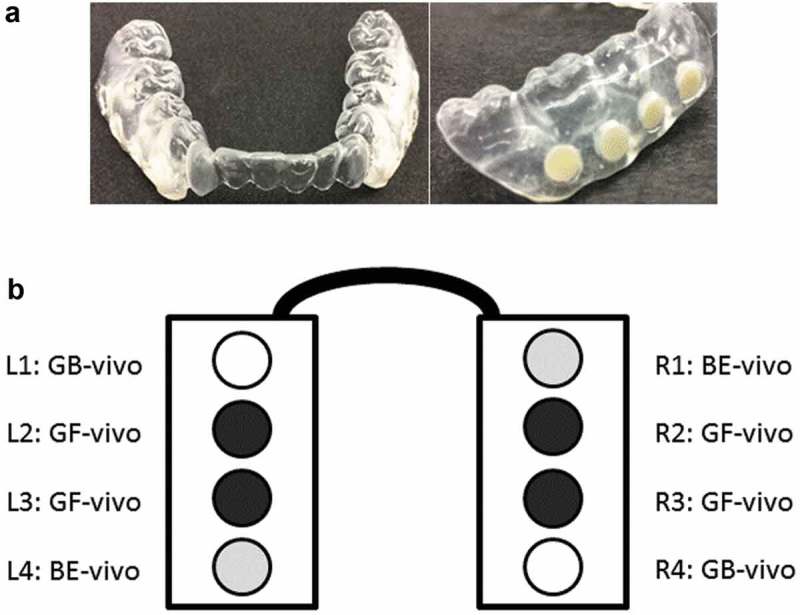
Intraoral appliances used in this study. Specimens were inserted next to the buccal region of the premolar and molar teeth. The samples were placed 1 mm below the plastic surface creating a retentive factor and therefore allowing plaque accumulation. (a) Appliance shown from mesial and vestibular (buccal) with incorporated test samples. (b) Sample distribution in oral appliances used for *in situ/in vivo* study. Bovine enamel (BE-vivo), Grandio Flow (GF-vivo), and Grandio Blocs (GB-vivo).

The 14 study volunteers were asked to wear a lower mandibular appliance composed of two composites (GF, GB) or BE as a control. Dental impressions of the lower jaw were used to produce thermoformed clear plastic retainers extended to the buccal area (Figure 1). Appliances were worn *in situ* for 48 h and kept in a box (with a humid atmosphere by adding a piece of wet tissue) only during meals and tooth brushing, the latter with a fluoride-free paste. After 48 h, the *in vivo* specimens were collected, and the presence of biofilms was confirmed by live/dead staining (Invitrogen FilmTracer Live/Dead Biofilm Vitality Kit, Thermo Fisher Scientific, Germany). All duplicate specimens were pooled in one extraction vial and stored as described below.

#### In vitro phase

Because carolacton is not yet approved for use in humans the comparison of GF and GF+C was carried out *in vitro*, using biofilms prepared from the saliva of the same volunteers enrolled in the *in vivo* study. We collected 6 ml of freshly paraffin-stimulated saliva from each participant and prepared microtiter plates containing four different test specimens (GF, GF+C, GB and BE) in duplicate for each volunteer, making 112 wells in total. To each well, we added 250 µl of saliva and 50 µl cell-free filtrate of the corresponding saliva sample as a nutritional source. The microtiter plates were sealed and incubated under strictly anaerobic conditions at 37°C for 48 h. Anaerobic conditions were chosen because previous studies have established that most oral bacteria grow when such conditions are selected [13,14].

After 48 h, the *in vitro* specimens were collected and biofilm-like structures were verified for a few representative specimens by live/dead staining as above. All duplicate disc specimens were pooled together in a 2-ml vial containing 200 µl DNA/RNA Shield medium (ZymoResearch, USA) plus 0.2 g acid-washed 0.1-mm zirconia-silica beads (Biospec, USA) and were stored at – 72°C.

### DNA sequencing and analysis

Biofilms were detached from the disc surface and disrupted by mechanical bead-beating using a FastPrep FP 120 (Qbiogene, USA) at 6.5 m/s for 45 s. DNA was extracted from *in vivo* biofilms (rich of extracellular polysaccharides but low cell numbers) using the MasterPure Complete DNA Purification Kit (Epicentre, USA) and from *in vitro* biofilms (less polysaccharides but high cell numbers) using the ZymoBIOMICS DNA Mini-Kit (ZymoResearch) to ensure optimal yields for standardized deep sequencing. Total DNA was prepared at the RWTH Division of Oral Microbiology and Immunology (Aachen, Germany) and sent on ice to the Helmholtz Center for Infection Research Next-generation Sequencing (NGS) Unit (Braunschweig, Germany) for library preparation and sequencing by V1-V2 primers 27F (5′-AGA GTT TGA TCM TGG CTC AG-3′) and 388R (5′-TGC TGC CTC CCG TAG GAG T-3′). Amplicon libraries for high-throughput sequencing on an Illumina MiSeq platform (280 bp paired-end chemistry) were prepared as previously described [15]. Quality filtering was applied to remove bad reads and chimeric sequences and data were analysed using the VSEARCH pipeline v2.6.0 [16], and the databases RDP [17] and HOMD [18] with OTU clustering at a 97% sequence identity threshold. OTUs with a total count <10 were eliminated. For the remainder, the *R*-based pipeline Rhea v1.6 was used for downstream normalization and the calculation of α-diversity (within samples), β-diversity (between samples) and taxonomic composition [19]. Bacterial diversity was presented by Shannon, Chao1 and Simpson indices. Taxonomic differences at the phylum, class, order, family, genus, species and OTU levels were compared among the study groups. Significant differences based on the prevalence among the different groups were calculated using permutation multivariate analysis of variance (PERMANOVA), Kruskal-Wallis rank sum test and Fisher’s exact test. The statistical significance threshold was set at p ≤ 0.05. The raw sequences were deposited at the European Nucleotide Archive (ENA) under the accession no. PRJEB31506.

## Results

### Sequencing and broad microbial profiles

The *in vitro* (GF, GF+C, GB and BE) and *in vivo* (GF, GB and BE) biofilm samples from probands P1–P14 initially yielded 98 samples in total for sequencing, producing a total of 7,362,488 reads, equivalent to a mean of 75,127 and a median of 77,342 reads per sample (range 1,296–111,348). One sample (P14, *in vivo*, GB) was excluded due to the loss of either the biofilm or DNA, and four others (P1, P5, P11, P14, all *in vitro*, all BE) due to read numbers below the post-rarefaction threshold of 20,000. The study data were therefore derived from 52 *in vitro* and 41 *in vivo* biofilm samples. The *in vitro* samples yielded a mean of 78,461 and a median of 87,672 reads (range 25,774–111,348) whereas the *in vivo* samples yielded a mean of 70,682 and a median of 71,999 reads (range 57,634–90,770).

Clustering, principal component analysis (PCA) and non-metric multidimensional scaling (NMDS) analysis of β-diversity revealed that most differences between microbiomes were conditional (*in vivo* versus *in vitro*) and/or individual-dependent, but were not related to the substrate material (**Supplementary Figure S1**). Interestingly, there was no significant difference in the *in vitro* NGS genotypic profiles of samples GF and GF+C. However, differences in the vitality (phenotype) of individual taxa were not investigated in this study.

**Figure 2.**
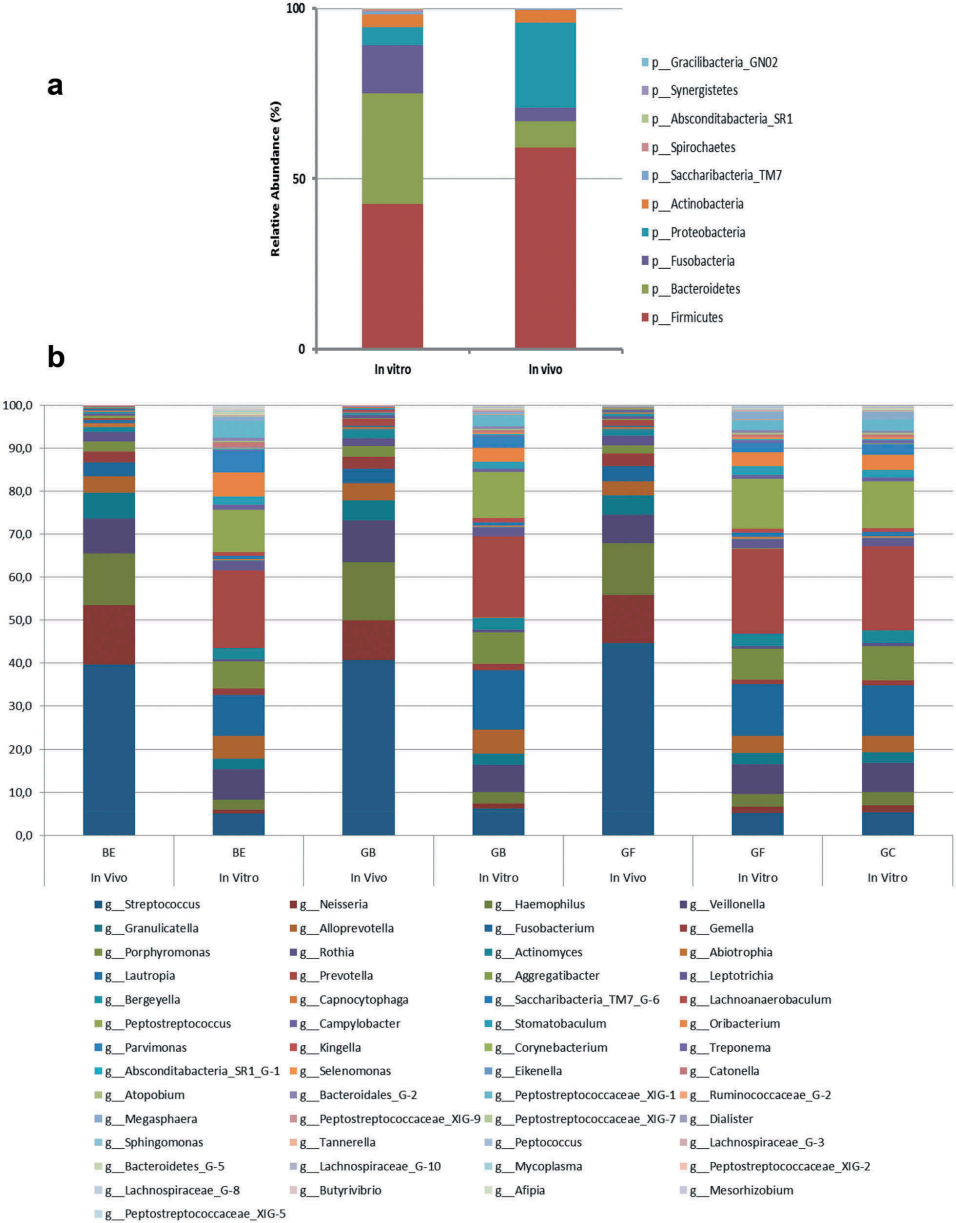
(a) Comparison of **phyla composition** of biofilm samples between *invitro* and *invivo* conditions: The phyla *Bacteroidetes* and *Fusobacteria* are more pronounced under strictly anaerobic *invitro* conditions. (b) Comparison of **genera composition** of biofilm samples on various composites (GB, GF, and GF+C abbreviated as GC) and under *in vitro* and *in vivo* conditions. Only genera of >1% relative abundance and presence on all materials were included. Data are mean values from 14 individuals. Please accept that for (a) and (b) a different colour scheme is used.

Sequencing revealed 10 different phyla in the *in vitro* samples, dominated by *Firmicutes* (mean 42.65%, median 42.81%), *Bacteroidetes* (32.5%, 32.36%), *Fusobacteria* (14.02%, 13.4%) and *Proteobacteria* (5.29%, 4.65%). We also identified 106 genera, principally *Prevotella* (19.06%, 20.21%), *Fusobacterium* (11.94%, 12.39%), *Peptostreptococcus* (10.79%, 10.17%), *Porphyromonas* (7.51%, 7.52%) and *Veillonella* (6.76%, 6.32%). The same 10 phyla were present in the *in vivo* samples, but their ranking was different, with *Firmicutes* (59.05%, 59.79%) followed by *Proteobacteria* (24.96%, 25.75%), *Bacteroidetes* (7.84%, 6.78%) and *Actinobacteria* (3.79%, 2.98%). We identified 97 genera, principally *Streptococcus* (41.69%, 41.71%), *Haemophilus* (12.49%, 10.62%), *Neisseria* (11.73%, 9.21%), *Veillonella* (8.10%, 5.41%) and *Granulicatella* (5.09%, 5.12%). These results are presented in Figure 2(a) (sorted by condition) and Figure 2(b) (sorted by material) with an overview in **Supplementary Figure S2**. The *in vitro* conditions clearly selected anaerobic taxa at the expense of aerobic or highly anaerobic taxa, the latter requiring redox potentials below – 110 mV which cannot be achieved using Gas Pak jars or similar systems. Some genera were found exclusively *in vitro* (*Bifidobacterium, Bosea, Leptothrix, Mesorhizobium, Microbacterium, Moraxella, Pedobacter, Peptoniphilaceae [G-3], Peptostreptococcaceae* [XI][G-1], *Peptostreptococcaceae* [XI][G-6], *Rhodocyclus* and *Turicella*) or *in vivo* (*Caulobacter, Mycobacterium* and *Sanguibacter*). However, these genera were only detected in 1–3 individuals and their relative abundance was very low. As all of these taxa are known residents of the human oral cavity (Human oral Microbial Taxa, HMTs) a contamination can be excluded so far as possible but not absolutely as there are many risks including those from reagents and kits [20]. It is important to notice that, because of the many differences between the *in vitro* and *in vivo* conditions and methods, a direct comparison of results is otherwise not warranted.

### In-depth analysis of the *in vitro* microbiome

**Figure 3.**
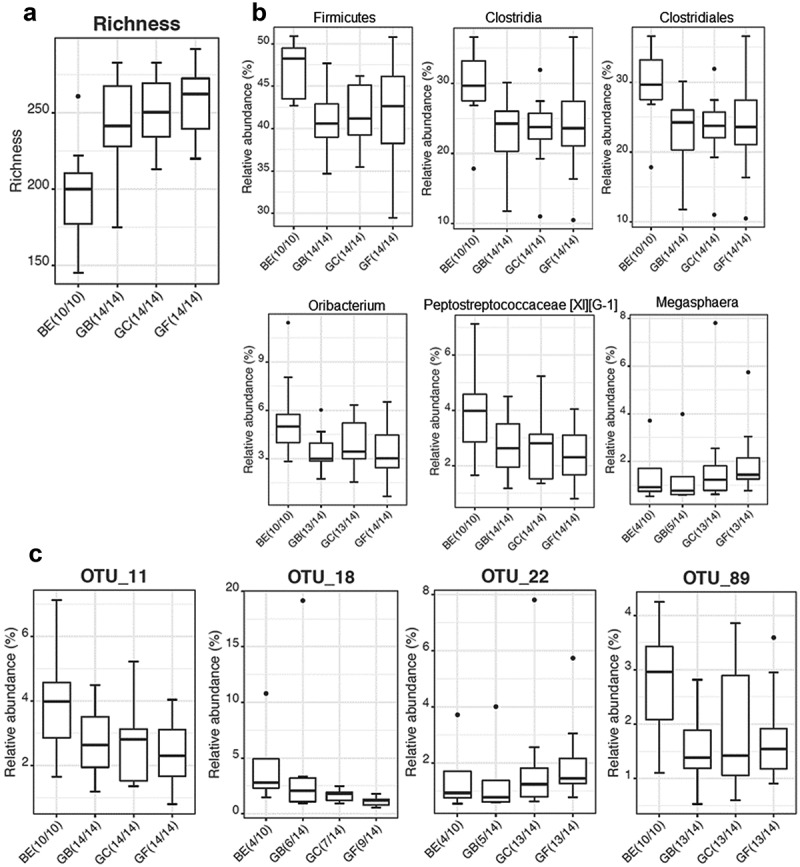
Taxa and OTUs grown ***in vitro*** of dominant bacteria (>1%) significantly different on bovine enamel versus various composites: (a) **Richness analysis**. The richness was significantly greater for all three composites compared to BE (Kruskal−Wallis Rank Sum Test − all groups p = 0.0264; Wilcoxon Rank Sum Test – pairwise GB vers. BE p = 0.0019, GF+C [abbreviated GC] vers. BE and GF vers. BE both <0.001) (b) **Taxon level**: *Firmicutes, Clostridia, Clostridiales* and the genera *Oribacterium* as well as *Peptostreptococcaceae* [XI][G-1] (‘*Eubacterium sulci’*) were found in higher and *Megasphaera* was found in significantly lower relative abundance on bovine enamel compared to composites (p = 0.0158); (c) **OTU Level** (similarity level >97%): OTU_11 (“*Eubacterium sulci*” | HMT_467 | strain ATCC 35585 like), OTU_18 (*Alloprevotella tannerae*, HMT_466 | ATCC 51259 like) as well as OTU_89 (*Oribacterium sinus* | HMT_457 | F0268-like) showed higher and OTU_22 (*Megasphaera micronuciformis* | HMT_122 | clone sequence) showed lower relative abundance on bovine enamel reaching significance level.

On a taxon and OTU level, the richness of the microbiome was significantly greater for all three composites compared to BE (Figure 3(a)). On taxon level, the phylum *Firmicutes* (p = 0.012), the class *Clostridia* (p = 0.015), the order *Clostridiales* (p = 0.015), and the genera *Oribacterium* (of the family *Lachnospiraceae*) (p = 0.048) and *Peptostreptococcaceae* [XI][G-1] (non-approved name: *Eubacterium sulci*) (p = 0.048) were significantly more abundant on BE than on the composites, whereas the genus *Megasphaera* showed the opposite profile (more abundant on composites GF+C and GF, p = 0.0002) (Figure 3(b)).

Among the OTUs with a relative abundance >1%, OTU_11 (*Eubacterium sulci*, Human oral Microbiome Taxon (HMT)_467, ATCC 35585-like), OTU_18 (*Alloprevotella tannerae*, HMT_466, ATCC 51259-like) and OTU_89 (*Oribacterium sinus*, HMT_457, strain F0268-like) were significantly more abundant on BE (p = 0.045) whereas OTU_22 (*Megasphaera micronuciformis*, HMT_122, clone sequence) showed the opposite profile (more abundant on composites GF+C and GF, p = 0.0002) (Figure 3(c)). These data revealed that *Clostridiales* of the genera *Oribacterium* and *Peptostreptococcaceae* [XI][G-1] preferred BE whereas members of genus *Megasphaera* favoured composites when cultivated anaerobically *in vitro*.

**Figure 4.**
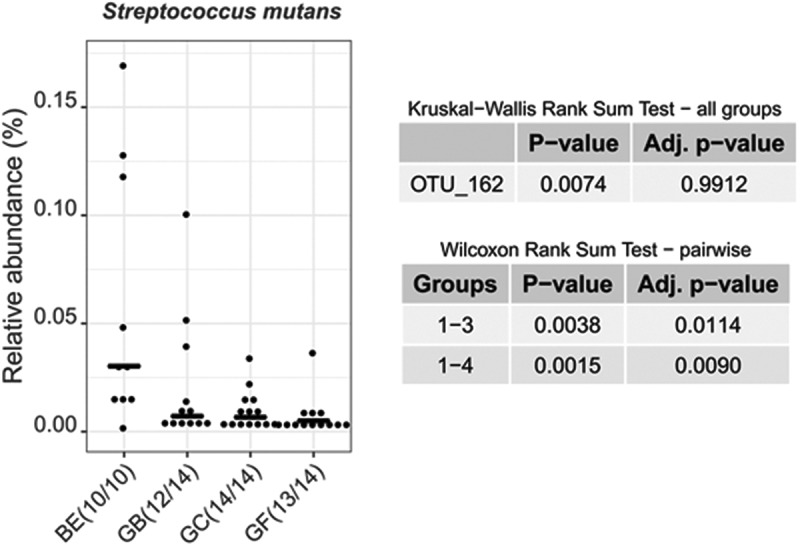
*Streptococcus mutans* relative abundance on four different substrates in vitro: Bovine enamel (BE), Grandio Blocs (GB), and Grandio Flow with (GF+C, abbreviated GC) and without carolacton (GF). The relative abundance is significantly higher comparing bovine enamel with Grandio Flow irrespective of releasing carolacton or not.

A few species with a low relative abundance (<1%) also grew (or at least attached) significantly better on composites than on BE: *Prevotella oulorum* (HMT_288), an unnamed *Streptococcus* species (HMT_431), *Veillonella atypica* (HMT_524), *V. parvula* (HMT_161), *Gemella morbillorum* (HMT_046) and *Fusobacterium periodonticum* (HMT_201) (**Supplementary Figure S3**). Interestingly, *Streptococcus mutans* was the only species that grew or attached significantly better on BE than on any of the composites (Figure 4). Among the 10 highest *S. mutans* counts among all specimens, eight were on BE *in vitro*. However, the relative abundance of *S. mutans* in our study was only 0.17%. The *S. mutans* identity and relative abundance was confirmed by the same qPCR as used for screening patients.

There were few differences in microbial profiles among the three different composites including GF+C. The relative abundance of OTU_22 (*M. micronuciformis*) and the genus *Megasphaera* was significantly higher on GF+C compared to GF (p = 0.0044) but also on GB compared to GF (p = 0.0044). No single OTU or taxon (including *S. mutans* and *S. mitis* HMT_677, the latter a close relative of *S. pneumoniae*) was significantly less abundant on GF+C.

### In-depth analysis of the *in vivo* microbiome

When comparing the different substrates *in vivo*, we observed no significant differences in richness and no significant differences in the prevalence of different taxa or OTUs among those with higher relative abundance. However, among the species with low relative abundance (<1%), *Granulicatella adiacens* was more prevalent on BE than on the composites and *Fusobacterium nucleatum* subsp. *animalis* showed the opposite behaviour (**Supplementary Figure S4**).

## Discussion

We investigated biofilm formation over 48 h on different composite materials compared to natural BE *in vivo* and *in vitro*, and also tested the effect of carolacton *in vitro* using a new biofilm model. Overall, we observed no significant difference after 48 h of bacterial colonization of the different dental composites and BE *in vivo*, confirming the negligible bacteriostatic/bactericidal effects of the composites against oral bacteria at low concentrations [21]. The richness of the microbiome was greater *in vitro* than *in vivo* (particularly on the composites) but this must be interpreted very carefully as the growth conditions *in vivo* and *in vitro* were very different. We did not observe any preferential growth of cariogenic bacteria on the composites compared to BE. Moreover, *S. mutans* tended to prefer enamel and/or the enamel-pellicle as an *in vitro* substrate perhaps reflecting positive tropism, disagreeing with many earlier reports showing the preferential adhesion and growth of *S. mutans* on dental composites (reviewed by Delaviz et al. 2014 [1]). However, we focused on the relatively early 48-h phase of biofilm formation and cannot rule out a compositional shift at later stages.

To the best of our knowledge, this is the first study comparing biofilm formation on composites and BE *in vivo* and *in vitro* using Illumina NGS technology [22]. A study using Roche 454 pyrosequencing revealed substrate-related differences in the diversity of *in vitro* biofilms during the initial phase of biofilm maturation, but the resins were modified with AgVO_3_ [23]. Biofilms on glass-ionomer cements were shown to be less diverse than those on resin composites and amalgam by denaturing gradient gel-electrophoresis and sequencing [24]. Many studies have used NGS to analyse the oral microbiome but have not considered the influence of restorations [25].

The incorporation of carolacton into GF did not show any effect *in vitro*. We previously incorporated 25 µg/ml carolacton (0.002%, thus half the concentration used in the present study) into GF and reduced the viability of *S. mutans* biofilms by 52–57% on day 1 decreasing slightly to 45% on day 42 [11]. That study demonstrated the release of carolacton from composites in effective concentrations over weeks. Here, we found that carolacton did not change the composition of the oral microbiome and thus is unlikely to reduce secondary caries. If we exclude technical issues such as inactivation during incorporation into the composite material, the lack of activity may reflect the biodegradation of carolacton, its sequestration by matrix components or resistance against its mechanism of action. Carolacton was recently shown to specifically inhibit the essential folate-dependent enzyme FolD/MTHFD, which is present in all bacteria [26]. However, in direct tests, carolacton has only been shown to inhibit certain isolates of *S. pneumoniae* [27] and does not affect the growth of most bacteria, including *S. mutans* at neutral or higher pH [10]. Resistance mechanisms have evolved such as the alternative enzyme Fhs, which is especially widespread in anaerobic bacteria [28], and the efflux pumps which most bacteria possess [26,27,29]. The strong effect of carolacton on *S. mutans* biofilm viability was always striking because it only occurs at low pH [30], reflecting the essential role of FolD in acid survival of *S. mutans* [31]. Our data indicate that a normal complex oral microbial community evades the inhibition of FolD by carolacton, most likely through the resistance mechanisms described above.

To establish a clinically relevant test model *in vitro*, we attempted to maintain saliva-derived biofilms *ex vivo* for 48 h without altering their *in vivo* composition using a combination of anaerobic incubation and feeding with salivary filtrate. We found that the microbial composition changed significantly *ex vivo* because the *in vivo* atmosphere and nutritional environment are difficult to reproduce. Nevertheless, our model has advantages over those in current use due to its intrinsic complexity. In contrast, most current models are based on a single species (usually *S. mutans, Porphyromonas gingivalis* or *Aggregatibacter actinomycetemcomitans*) or communities of up to 12 species, allowing the investigation of primary or secondary caries [32–34] or the efficiency of antimicrobials and disinfectants [35–37]. The Zürich model with five bacterial species (plus/minus *Candida albicans*) is one of the most widely used [35]. Recently, an *in vitro* biofilm was grown from the saliva of a single donor for 48 h, to study the diffusion of antimicrobials in oral biofilms [13]. This is similar to our model but the biofilm was grown in narrow (100 μm) channels and not on composite or BE specimens. Furthermore, oral biofilms initiated from the saliva of a single donor have been grown anaerobically in basal medium mucin for up to 10 days and used to investigate changes in the microbiome by 16S rDNA gene amplicon sequencing of 10 species [14]. A complex *in vitro* biofilm initiated from the subgingival plaque of patients with periodontal disease was grown in trypticase soy broth with several re-inoculation cycles to analyse the community structure by DNA-DNA checkerboard hybridisation covering seven species [38]. Several animal models have also been used to study antimicrobial activity against biofilms although the microbial communities differ substantially from those in humans [39,40].

Our new approach provided insight into the microbial communities during 48 h of biofilm formation but has some limitations. First, the difference in β-diversity between *in vitro* and *in vivo* biofilms was exacerbated by using two different DNA extraction methods to ensure optimal yields for standardized deep sequencing, although both involved an initial mechanical lysis step. Second, all enrolees were characterized by a low relative abundance of *S. mutans* (≤0.17%), and it would be interesting to test individuals with a more prevalent *S. mutans* population. Third, we used BE rather than the more relevant human enamel due to the need for a large number of equally sized high-quality specimens. Fourth, the pooling of two discs might have masked the variability between sites. Fifth, the V1-V2 16S rRNA primers were chosen to facilitate the identification of streptococcal species, which may have biased the diversity of the resulting microbial composition [41]. Finally, the standard OTU clustering at a 97% sequence identity threshold might need re-consideration for accuracy according to Edgar 2018 [42], and we recommend to use a 98.7% threshold or higher [15].

In conclusion, other than a few exceptions, we found that the oral microbiome does not change with respect to genera/OTU’s when grown on BE or on various composites, even in the presence of carolacton, possibly ruling out the promotion of particular cariogenic flora *per se* as the reason for elevated secondary caries around composite restorations. Our new *ex vivo* biofilm model was shown to keep most of the *in vivo* bacterial taxa but still major differences in the bacterial composition occurred, mainly due to the selection of a defined atmosphere which does not reflect the complex atmospheric conditions (aerobic, microaerobic, anaerobic together in proximity) in the oral cavity. However, this model might still be useful for the pre-clinical testing of preventive strategies.
