# Supplementary material for: Deep sequencing of biofilm microbiomes on dental composite materials
Source: J Oral Microbiol. 2019 May 14;11(1):1617013. doi: 10.1080/20002297.2019.1617013 (PMC6522937; doi:10.1080/20002297.2019.1617013)
Supplement: Supplemental Material [file ZJOM_A_1617013_SM8852.docx]

Supplementary:

Deep sequencing of early biofilm microbiomes on dental composite materials

**
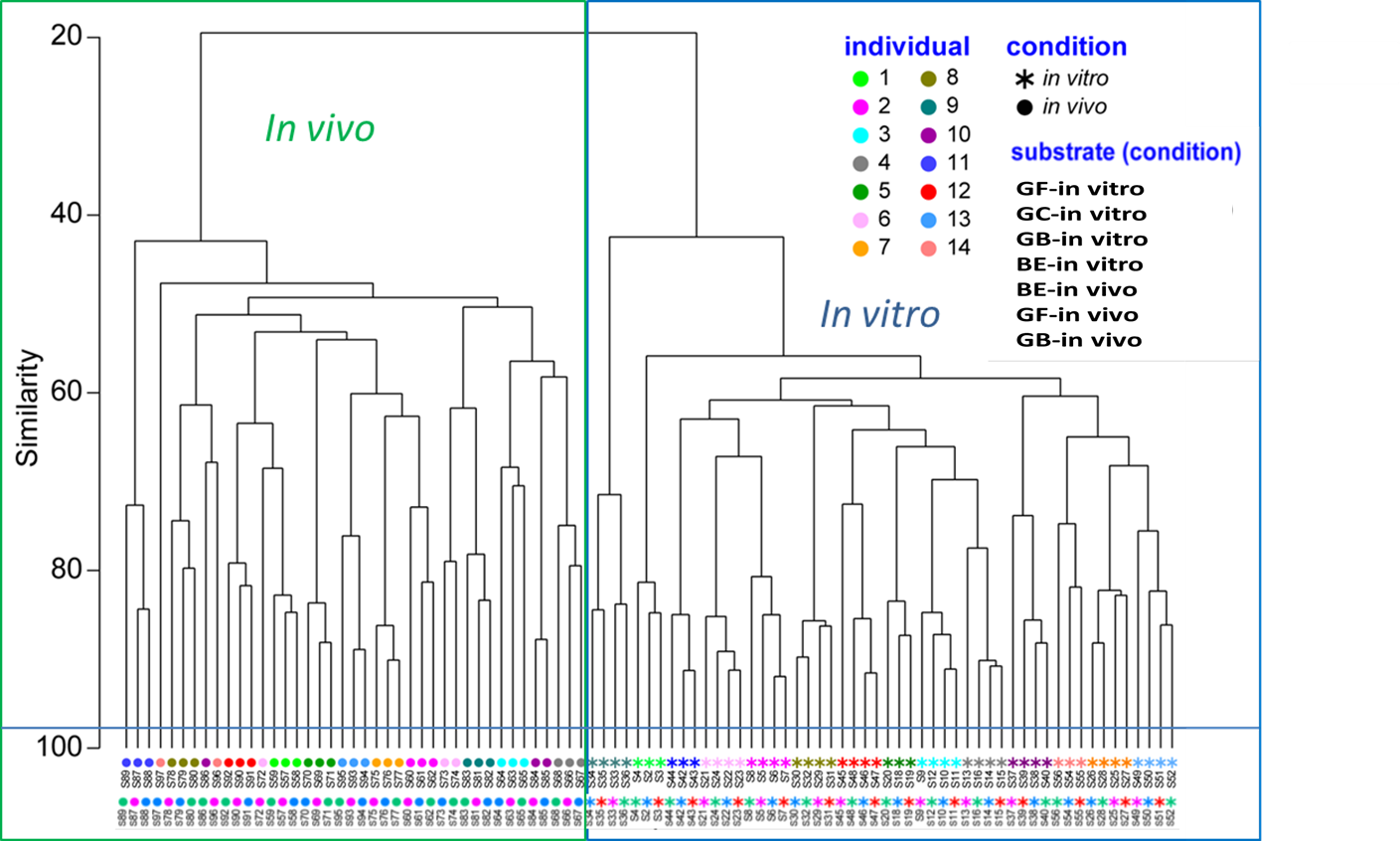
**

**A**

**
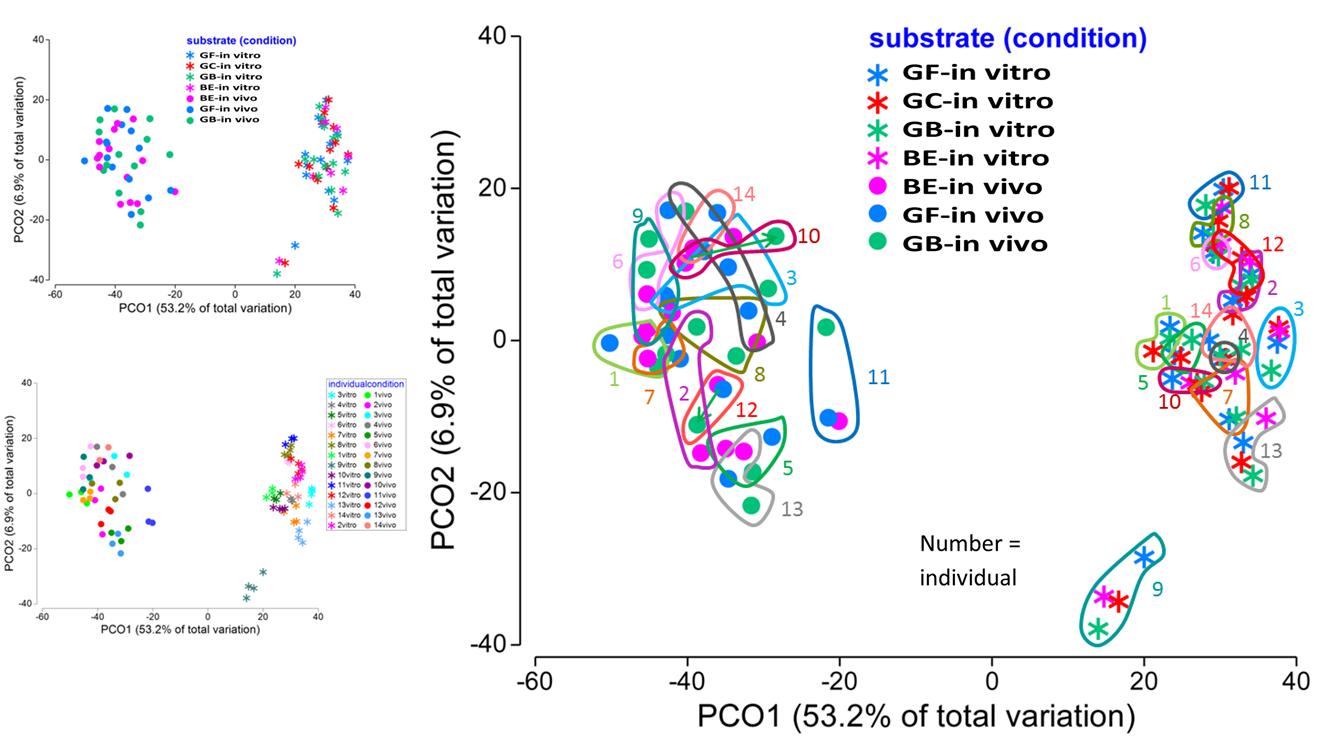
**

**B**

**C**

**In vitro**

**In vivo**

**Supplementary Figure S1.** Beta-diversity of biofilm microbiomes grown on substrates subjected in this study **(A)** Cluster Analysis: results are labelled by condition (in vivo versus in vitro), substrate (BE, GC, GF, GB), and individual (1-14). Clearly, the clustering is influenced (in the order of importance) by i) condition, ii) individual (biofilm donor), and – only partially - iii) substrate. **(B)** Principal component analysis: results show the same hierarchy of parameter importance: condition > individual > substrate. **(C)** Non-metric multidimensional scaling (NMDS): over all taxa and OTUs, the beta-diversity is not significantly different on the various substrates: Bovine enamel (BE), Grandio Flow (GF), Grandio Flow with carolacton (GC, in vitro only) and Grandio Blocs (GB).


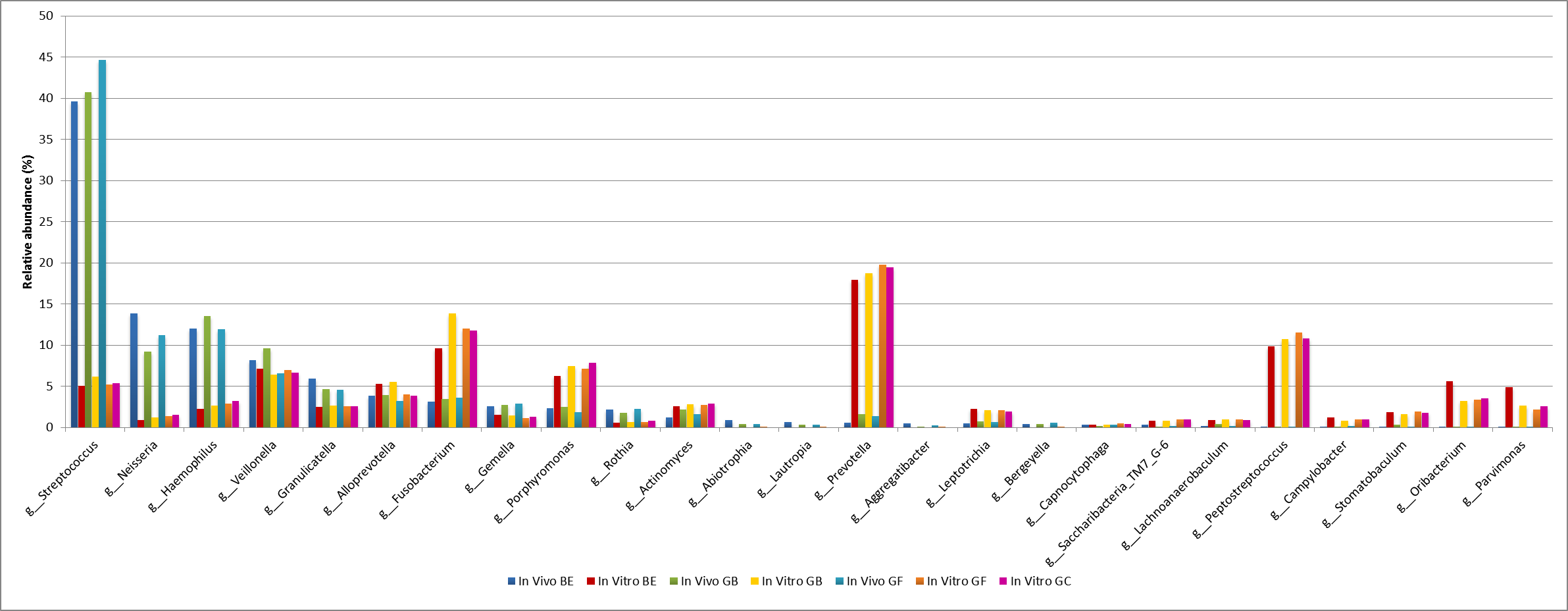


**Supplementary Figure S2.** Comparison of genera composition of biofilm samples on bovine enamel (BE) and on various composites (GB, GF, GC) under in vitro (blue-green-turquoise) and in vivo (red-yellow-orange-magenta) conditions. Only genera of >0.04% relative abundance and presence on all materials were included. Data are mean values from 14 individuals. The genera *Streptococcus, Neisseria, Haemophilus*, *Granulicatella*, *Gemella*, and *Rothia* dominate in the in vivo phase while *Fusobacterium, Porphyromonas, Prevotella, Leptotrichia, Peptostreptococcus , Campylobacter, Stomatobaculum, Oribacterium* , and *Parvimonas* were obviously supported by the strictly anaerobic in vitro conditions.

**Supplementary Figure S3.** OTUs of biofilm grown **in vitro** of general low abundance (<1%) significantly different on bovine enamel compared to various composites: OTU-162 (*Streptococcus* *mutans*) was the only species with a higher relative abundance on enamel while a few species showed higher relative abundances on composites, namely OTU_61 (*Prevotella oulorum* | HMT_288 | strain_ATCC 43324), OTU_104 (unnamed *Streptococcus* sp. | HMT_431 | Clone_C4AKM023), OTU_346 (*Veillonella atypica* | HMT_524 | DSM 20739), OTU_416 (*Veillonella parvula* | HMT_161 | DSM 2008), OTU_550 (*Gemella morbillorum* | HMT_046 | 933-88), and OTU_1754 (*Fusobacterium periodonticum* | HMT_201 | ATCC 33693).

**Supplementary Figure S4.** OTUs grown **in vivo** of general low abundance (<1%) significantly different on bovine enamel compared to various composites: OTU-861 (*Granulicatella adiacens* | HMT_534 | Strain_TKT1) was the only species with a higher relative abundance on enamel while OTU_425 (*Fusobacterium nucleatum* subsp. *animalis* | HMT_420 | NCTC 12276) showed higher relative abundances on composites.
